# Study of Silver Nanoparticles Sensitized Fluorescence and Second-Order Scattering of Terbium(III)-Pefloxacin Mesylate Complex and Determination of Pefloxacin Mesylate

**DOI:** 10.1155/2014/742935

**Published:** 2014-05-06

**Authors:** Aiyun Li, Zhiqiang Song

**Affiliations:** ^1^Experimental Center of Fundamental Theories, Shandong Sport University, Jinan 250102, China; ^2^Department of Physical Education, Shandong Sport University, Jinan 250102, China

## Abstract

*α*-Keto acid of pefloxacin mesylate (PFLX) can form the complex with Terbium(III). The intramolecular energy from PFLX to Terbium(III) ion takes place when excited, and thus Terbium(III) excited state is formed and then emits the characteristic fluorescence of Terbium(III), locating at 490, 545, 580, and 620 nm. The second-order scattering (SOS) peak at 545 nm also appears for the complex with the exciting wavelength of 273 nm. When the silver nanoparticles are added to the system, the luminescence intensity at 545 nm greatly increased. So, with the adding of nanoparticles to the Terbium(III)-PFLX complex, not only is the intramolecular energy promoted but also the SOS intensity is enhanced. The experimental results show that it is the silver nanoparticles with certain size and certain concentration which can greatly enhance the fluorescence-SOS intensity, and the relative intensity at 545 nm is proportional to the amount of PFLX. Based on this phenomenon, a novel method for the determination of PFLX has been developed and applied to the determination of PFLX in capsule and serum samples.

## 1. Introduction

In recent years, researches on noble metals nanoparticles have got considerable attention in chemistry and physics [1–3] especially silver nanoparticles, which exhibit an enhancement of some potential properties including electrical conductivity [4], catalysis [5, 6], magnetic and optical polarizability [7], photonic technologies [8–11], and antimicrobial activity in surface-enhanced Raman scattering (SERS) [12]. Particle size may influence the physical properties of silver nanoparticles [13–15]. With the progress of nanotechnologies and the spectrum theories, more studies have been attracted to the luminescence of the role of silver nanoparticles [16, 17].

Terbium ions have unique fluorescent properties when complexed with organic ligands. The strong ion emission of these complexes originates from an intrachelate energy transfer from the triplet state of the ligand to the excited energy levels of the lanthanide ion. Methods for the determination of several organic compounds, which serve as energy donors to Terbium ions, have been developed [24].

**Figure 1:**
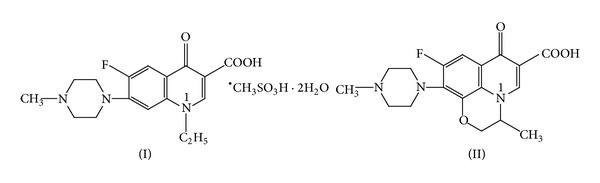
Structural formulae of the PFLX (I) and OFLX (II).

Ofloxacin (OFLX), (±)9-fluoro-2,3-dihydro-3-methyl-10-(4-methyl-1-piperaziny)-7H-oxo-7H-pyrido[1,2,3-de]-1,4-benzoxazine-6-carboxylic acid (as shown in Figure 1), is one of the third-generation members of quinolone synthetic antibiotics, with a broad spectrum of activity against Gram-positive and Gram-negative bacteria [25, 26]. It is widely used in therapies against inflammation [27]. The drug's effect is concentration dependent and its antibacterial effect is closely related to its plasma concentration.

Pefloxacin mesylate (PFLX), 1-ethyl-6-fluoro-1, 4-dihydro-7-(4-methylpiperazin-1-yl)-4-oxoquinolone-3-carboxylic acid (as shown in Figure 1), is a fluoroquinolone antibacterial agent. It is used for the treatment for diseases of the skin and various kinds of urinary tract infections [28] and has been widely applied in clinical medicine. Determination methods for PFLX include spectrofluorometry [24, 29, 30], HPLC [31], TLC-fluorescence [32], and capillary electrophoresis [33, 34]. But using the silver nanoparticlessensitized fluorescence and second-order scattering (SOS) for the determination of PFLX has not been reported.

In this paper, the influence of silver nanoparticles on the SOS and fluorescence of Terbium(Tb)(III)-PFLX complex is studied through using the SOS and fluorescence spectrum. The results show that the size and concentration of nanoparticles can greatly affect the fluorescence-SOS intensity of the complex. Based on this phenomenon, a novel method has been developed for the determination of PFLX, and the determination comparison between PFLX and OFLX is also proposed.

## 2. Material and Methods

### 2.1. Apparatus

A transmission electron microscopy (TEM) image of the silver nanoparticles was acquired using a Hitachi H-600 (Japan) transmission electron microscope. A Shimadzu RF-5301 PC spectrofluorometer (Japan) was used for fluorometric and SOS measurements. All absorption spectra were recorded with a Cintra 10e UV-vis spectrophotometer (GBC). A 420A plus pH meter (Orion Research Inc) is used to measure pH of the solutions. All reagents were of analytical reagents grade and doubly distilled water was used throughout.

Stock standard solution (1.0 × 10^−3^ M) of PFLX and OFLX (Institute of Medical Biotechnology, Beijing, China) was prepared by dissolving them in proper solvent (dilute acid or alkali) and then was diluted to the desired concentration with water.

### 2.2. Reagents

A standard stock solution of the Tb(III) ion (1.0 × 10^−2^ M) is prepared by dissolving 934.5 mg Tb_4_O_7_ in 15 mL HCl (12 M) at 100°C and evaporating the solution to be almost dry; it is then diluted to 500 mL with water.

**Figure 2:**
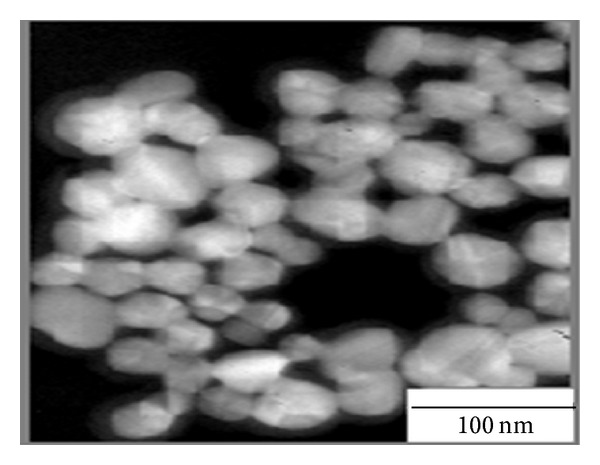
TEM image of the silver nanoparticles.

The preparation of the silver colloids followed the same procedure as originally proposed by Lee and Meisel [35]. AgNO_3_ is used as precursor of silver nanoparticles and sodium citrate is used as both reducing and protecting reagent. A concentration of 1.0 × 10^−4^ M colloidal solution is prepared in terms of the silver atoms. The morphology and size distribution of silver nanoparticles are obtained by a transmission electron microscopy (TEM) and were shown in Figure 2. The particles are almost spherical with a mean diameter of 42 nm, and the silver nanoparticles had an absorption maximum at 420 nm, which is consistent with the literature [35].

The drug content of five capsules is weighed, finely powdered, and mixed. The average mass per capsule can be determined. Transfer an accurately weighted amount of the powder equivalent to 200 mg corresponding to one capsule into a 100 mL calibrated dark flask, in which the deionized water is added to dissolve the powder. The solution is then filtered so as to separate out the insoluble excipients. The desired concentration for the drug thus is obtained by accurate dilution with deionized water and the analysis is followed up as the general analytical procedure.

### 2.3. Procedures

As for a 10 mL test tube, 1.0 mL of HAc-NaAc buffer solution, 0.2 mL of 1.0 × 10^−2^ M Tb(III) ion solution, and an appropriate working solution or sample solution of PFLX, followed by 0.5 mL of 1.0 × 10^−4^ M silver nanoparticles are added. This mixture is diluted to 10 mL with water, mixed thoroughly, and stood for 25 min.

The SOS intensity is recorded with the different excited wavelength from 220 to 400 nm and reached the maximum at 545 nm with *λ*_ex_ = 273 nm. The enhanced SOS and fluorescence intensities are represented as Δ*I* = *I* − *I*0; here, *I* and *I*0 are the intensities of the system with and without PFLX or OFLX. All the data are obtained with 5.0 nm excitation and emission silt-widths.

## 3. Results and Discussion 

### 3.1. UV-Vis Spectrum

**Figure 3:**
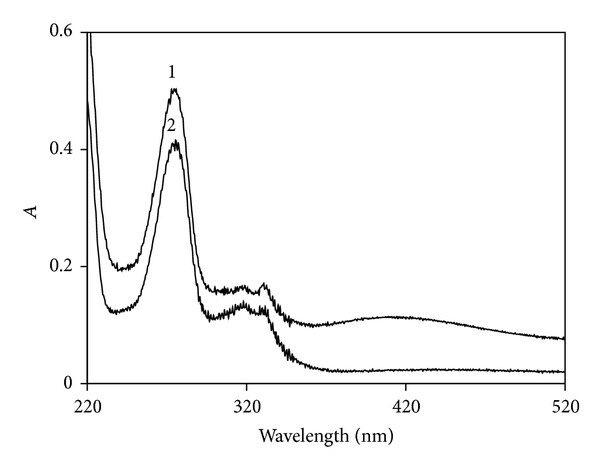
UV-vis absorption spectra. (1) Silver nanoparticles-Tb(III)-PFLX; (2) Tb(III)-PFLX. Conditions: silver nanoparticles, 5.0 × 10^−6^ M; Tb(III), 2.0 × 10^−4^ M; PFLX, 1.0 × 10^−6^ M.

UV-vis absorption spectra of the system (Figure 3) are recorded. It can be found that the absorption peaks at 275 nm and 323 nm for Tb(III)-PFLX complex increased along with adding silver nanoparticles to the system, and the absorption peak at 420 nm for the nanoparticles appeared. The results indicate that there exist interactions between Tb(III)-PFLX and silver nanoparticles and it can be concluded that silver nanoparticles incorporate with the complex of Tb(III)-PFLX, while the particle aggregates are formed in the ternary complex [16].

### 3.2. Second-Order Scattering Spectra and Fluorescence Spectra

**Figure 4:**
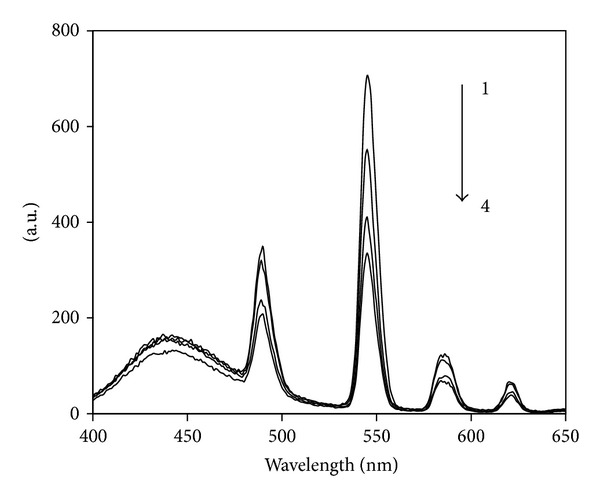
The fluorescence and second-order scattering spectra. (1) Silver nanoparticles-Tb(III)-PFLX (without filter), (2) silver nanoparticles-Tb(III)-PFLX (with filter), (3) Tb(III)-PFLX (without filter), and (4) Tb(III)-PFLX (with filter); *λ*_ex_ = 273 nm. Conditions: silver nanoparticles, 5.0 × 10^−6^ M; Tb(III), 2.0 × 10^−4^ M; PFLX, 1.0 × 10^−6^ M.

*α*-Keto acid of PFLX supplies a coordination site binding to Tb(III) ion and has two aromatic rings that can absorb energy, resulting in intramolecular energy from PFLX to Tb(III) ions; thus, Tb(III) excited state is formed and then emits the characteristic emission of Tb(III), locating at 490, 545, 580, and 620 nm, corresponding to the transitions of the Tb(III) 5D4 → 7F6, 5D4 → 7 F5, 5D4 → 7F4, and 5D4 → 7F3, respectively [36]. The maximum fluorescence peak locates at 545 nm when it is excited; the silver nanoparticles can enhance the fluorescence of Tb(III)-PFLX complex, especially at 545 nm. At the same time, the SOS peak is also enhanced at 545 nm when the excited wavelength is at 273 nm, so the intensities conclude the fluorescence and SOS intensity.  Figure 4 shows the fluorescence and SOS spectra, and the UV-35 filter is added to eliminate the scattering of the system [37].

### 3.3. Comparison between Silver Nanoparticles-Tb(III)-PFLX and Silver Nanoparticles-Tb(III)-OFLX System

**Figure 5:**
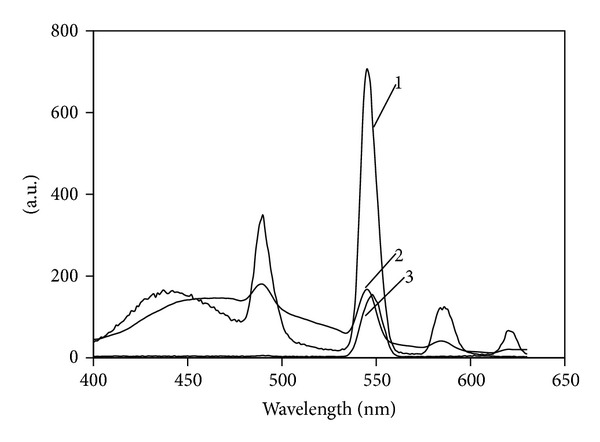
The fluorescence and second-order scattering spectra of PFLX and OFLX. (1) Silver nanoparticles-Tb(III)-PFLX, (2) silver nanoparticles-Tb(III)-OFLX, and (3) silver nanoparticles-Tb(III). Conditions: silver nanoparticles, 5.0 × 10^−6^ M; Tb(III), 2.0 × 10^−4^ M; PFLX and OFLX, 1.0 × 10^−6^ M.

From the fluorescence-SOS spectra above, the difference between silver nanoparticles-Tb(III)-PFLX and silver nanoparticles-Tb(III)-OFLX system could be observed (as shown in Figure 5). Silver nanoparticles can enhance the fluorescence-SOS intensity at 545 nm of Tb(III)-PFLX complex, while it can slightly increase the intensity of Tb(III)-OFLX system.

The observed spectral differences seem to be dependent on the structural variation of the fluoroquinolone and especially on the nature of the N1 substituent. The benzoxazine group of OFLX appears as an efficient electron-attracting system acting as a quencher of the fluorescence process. In contrast, the PFLX system has strong intensity owing to the electron-donating character of the N1 ethyl substituent [38]. The molecular structures of PFLX and OFLX are shown in Figure 1.

**Figure 6:**
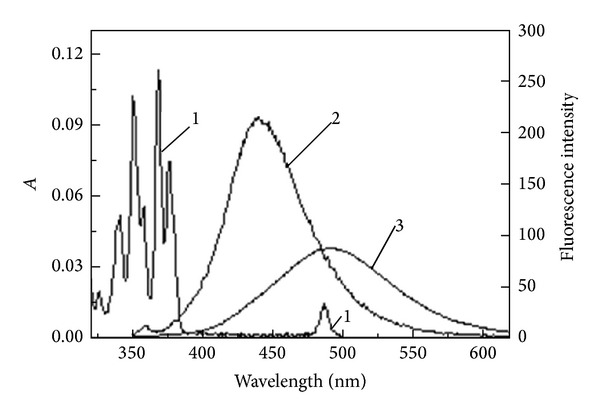
Absorption spectrum of Tb(III) (1) and emission spectra of PFLX (2) and OFLX (3) Conditions: Tb(III), 2.0 × 10^−4^ M; PFLX and OFLX, 1.0 × 10^−6^ M.

On the other hand, according to the theory of “the trivial of radiative mechanism for electronic energy transfer” [39], the efficiency of energy transfer is related to the capability of absorbing photon (*ε*) for donor and the overlap of the emission spectrum of donor and the absorption spectrum of acceptor. In Tb(III)-PFLX and Tb(III)-OFLX complexes, *ε*_PFLX_ > *ε*_OFLX_, at the same time, the emission spectrum of PFLX has a larger overlap with the absorption spectrum of Tb(III) than that of OFLX (see Figure 6); thus the efficiency of energy transfer of Tb(III)-PFLX system is much higher than that of Tb(III)-OFLX system, resulting in notable difference between them when the silver nanoparticles are added.

### 3.4. Influence of pH

Fluorescence and SOS intensity of the system is pH dependent. The effect of pH was investigated over the range of 3.0–8.0, and the optimum pH is about 6.0. Then a HAc-NaAc buffer solution of pH 6.0 with a concentration of 0.1 M was found to be suitable for the measurements. Maybe the reason is that Ac^−^ replaces H_2_O which quenches fluorescence of the Tb(III) complex and incorporates with Tb(III)-PFLX complex [40].

### 3.5. Effect of Tb(III) Ion Concentration

The effect of Tb(III) ion concentration in the range from 1.0 × 10^−5^ to 5.0 × 10^−4^ M on the analytical signal for the system was studied. As the concentration of Tb(III) ion increased, the relative intensity was enhanced; when the concentration was over 2.0 × 10^−4^ M, the relative intensity remains constant. So a Tb(III) ion concentration of 2.0 × 10^−4^ M was selected for further research.

### 3.6. Effect of Silver Nanoparticles Concentration

From the experiments, it can be inferred that not only the size but also the concentration of nanoparticles (namely, the numbers of particles in the unit volume) can affect the luminescence intensity of the system. The silver nanoparticles are prepared by AgNO_3_ solutions of 1.0 × 10^−3^ M, 1.0 × 10^−4^ M, and 1.0 × 10^−5^ M, respectively. The results show that if AgNO_3_ was 1.0 × 10^−3^ M, the silver nanoparticles have very high SOS scattering and the relative intensity is not proportional to the concentration of drug; if AgNO_3_ is 1.0 × 10^−5^ M, the scattering intensity of silver nanoparticles was very low and the fluorescence-SOS could not be enhanced by the silver nanoparticles. The silver nanoparticles are suitable for this system if AgNO_3_ is 1.0 × 10^−4^ M and the mean diameter is 42 nm, and thus they are chosen in the experiments.

Fixing the size of silver nanoparticles mentioned above, the effects of the concentration on the intensity of SOS and fluorescence are also studied. A concentration of 5.0 × 10^−6^ M of silver nanoparticles is used for further experiments.

## 4. Stability

Under the optimum conditions, the results showed that Δ*I* reached a maximum after all reagents were mixed for 25 min and the intensity was stable for at least 3 h, so that 25 min was set as the standard for all the SOS and fluorescence measurements.

### 4.1. Tolerance of Foreign Substances

In order to assess the possibility of analytical application of the method, the effects of some common excipients, metal ions, and organic compounds on signals intensity are investigated. The tolerable concentration ratios for the interference are <±5%. The results are presented in Table 1. It can be seen from Table 1 that most of metal ions can be allowed at higher concentrations, but some organic species such as myoglobin, Vitamin B1, and uric acid have a relatively high interference. In sample determinations, starch and dextrin which exist in capsules can be eliminated through filtration, while in the serum samples, ZnSO_4_ and Ba(OH)_2_ can be added in order to precipitate the interference before the determination [40]. So it can be successfully applied to determine PFLX in capsules and serum samples.

### 4.2. Calibration and Detection Limitation

The calibration graphs for the determination of PFLX are conducted under the optimal conditions, and the results are given in Table 2. The detection limit (3*σ*) is 2.5 × 10^−10^ M for PFLX.

### 4.3. Samples Determination

The proposed method is applied to the determination of PFLX in capsules and compared with UV-vis method; the results are given in Table 3. There are no significant differences between labeled contents and those obtained by this method. Recoveries range from 98.8% to 104.0%.

Moreover, analytical recoveries were assessed by analyzing serum samples which contain PFLX and required only separation of the precipitated protein with centrifugation [41]. In order to make the sample concentrations of the drug within the linear range of the determination, serum samples were properly diluted and the recoveries were determined by the standard addition method [42]. The results are shown in Table 4.

## 5. Conclusion

The proposed silver nanoparticles sensitized fluorescence and SOS method for the determination of PFLX is simple, rapid, and could be easily automated. At the same time, this method shows high sensitivity and wide linear response for the determination of PFLX; the sensitivity of this method is higher than that of most other methods summarized in Table 5. Since nanoparticles have unique physical and chemical properties, applications of nanoparticles in analytical chemistry are very potential. But the mechanism needs further study.

## Figures and Tables

**Table 1:** Tolerance of coexisting substance^a^.

Substance	Concentration coexisting (×10^−6^ M)	Intensity change (%)	Substance	Concentration coexisting (×10^−6^ M)	Intensity change (%)
Hemoglobin	10^b^	+0.4	Fe^3+^, NO_3_^−^	10	+3.4
Myoglobin	1^b^	−0.1	Mg^2+^, SO_4_^2−^	1000	+2.4
Vitamin B1	5^b^	−4.7	NH_4_^+^, Cl^−^	10000	+4.5
Glucose	500^b^	+4.8	K^+^, Cl^−^	1000	−1.3
Fructose	500^b^	+4.4	Co^2+^, SO_4_^2−^	100	−1.1
Starch	100^b^	+4.0	Na^+^, Cl^−^	10000	+4.6
*β*-alanine	100^b^	−0.7	Mn^2+^, SO_4_^2−^	1000	+4.8
*β*-CD	100^b^	+2.7	Cd^2+^, Ac^−^	100	−3.2
Uric acid	5	−2.6	Zn^2+^, SO_4_^2−^	100	−0.7
*β*-dextrin	50	+1.2	Pb^2+^, NO_3_^−^	100	+1.9
Al^3+^, SO_4_^2−^	10	+5.1	K^+^, H_2_PO_4_^−^	10	+4.1
Ni^2+^, NO_3_^−^	10	+3.0	Cr^3+^, Cl^−^	100	−4.0
Cu^2+^, SO_4_^2−^	10	−4.9	Ca^2+^, Cl^−^	1000	+4.8

^a^Conditions: PFLX, 1.0 × 10^−6^ M; Tb(III) ion, 2.0 × 10^−4^ M; silver nanoparticles, 5.0 × 10^−6^ M; pH, 6.0 × 10^−6^ g mL^−1^.

**Table 2:** Analytical parameters for the determination of PFLX.

	Linear range (×10^−7^ M)	Linear regression equation (*c* × 10^−7^ M)^a^	*r* ^ b^	LOD^c^ (3*σ*, ×10^−10^ M)
PFLX	0.008~10.0	Δ*I* = 7229.5*c* − 9.2828	0.9991	2.5
	10.0~80.0	Δ*I* = 4653*c* + 419.95	0.9973	

^a^Δ*I* is the enhanced intensity of SOS and fluorescence.

^b^Correlation coefficient.

^c^Detection limit.

**Table 3:** Results for the determination of PFLX in capsules (*n* = 5).

Sample	Labeled (mg)	Amount found ± R.S.D.%		Added (×10^−7^ M)	Found (×10^−7^ M)	Recovery ± R.S.D (%)
		This method	UV-method			
PFLX (capsule 1)	200.0	200.4 ± 3.6	197.2 ± 1.2	1.00	1.04	104.0 ± 3.60
				2.00	2.04	102.0 ± 2.90
				4.00	4.06	101.5 ± 2.92

PFLX (capsule 2)	200.0	200.2 ± 1.5	196.9 ± 3.9	6.00	6.10	101.7 ± 2.46
				8.00	8.20	102.5 ± 3.63
				10.00	9.88	98.8 ± 3.92

**Table 4:** Analytical recoveries of PFLX in serum samples (*n* = 5).

PFLX	Added (×10^−8^ M)	Found (×10^−8^ M)	Recovery ± R.S.D. (%)
Serum 1	1.00	1.03	103.0 ± 3.21
	2.00	2.03	101.5 ± 3.89
	3.00	3.02	100.7 ± 4.53

Serum 2	3.00	3.12	104.0 ± 3.96
	5.00	4.79	95.8 ± 2.43
	7.00	6.92	98.8 ± 3.36

**Table 5:** Common methods for the determination of PFLX.

probe	Limit of determination (3*σ*)	Method of detection	Reference
Water-soluble CdTe quantum dots	1.3 × 10^−8^ g/mL	Fluorescence	[18]
Terbium(III)	3.2 × 10^−9^ g/mL	Fluorescence	[19]
La(III)	2.8 × 10^−9^ g/mL	Fluorescence	[20]
Ce^4+^-Na_2_SO_3_-H_2_SO_4_	2.0 × 10^−8^ g/mL	Chemiluminescence	[21]
SDS-Terbium(III)	3.0 × 10^−9^ mol/L	Fluorescence	[22]
	(1 × 10^−9^ g/mL)		
Charge-transfer reaction	5.46 × 10^−6^ g/mL	Spectrophotometry	[23]
Silver nanoparticle-Terbium(III)	2.5 × 10^−10^ mol/L	Fluorescence and second-order scattering	This paper
	(0.8 × 10^−10^ g/mL)		
